# Opening Editorial: Selection and Recruitment in Medical Education

**DOI:** 10.15694/mep.2018.0000222.1

**Published:** 2018-10-01

**Authors:** Fiona Patterson, Barbara Griffin, Mark D Hanson

**Affiliations:** 1 Work Psychology Group; 2 Macquarie University; 3 University of Toronto

**Keywords:** Selection, recruitment, medical school admissions

## Abstract

This article was migrated. The article was marked as recommended.

There is over a century of research on selection and recruitment and the field has both developed and expanded significantly over this time. Previous research has tended to focus on reviewing the effectiveness of selection methods (academic records, references, personal statements, aptitude tests, personality assessments, situational judgement tests, and interviews), where good quality evidence is now emerging.

Many challenges remain however, reflecting that selection and recruitment into medical education (both undergraduate and postgraduate) is a complex, multi-dimensional, dynamic phenomenon. For example, issues regarding diversity and fairness in selection have been researched over many years but there remains a huge gap between the research evidence and policy enactment in many parts of the globe.

In this opening editorial for our special issue on selection and recruitment in medical education we encourage authors to consider six key question areas (amongst others), including:

• how will technology (e.g. social media, big data, artificial intelligence, etc) influence selection research and practices in future?
• should selection criteria be reviewed to include creativity, innovation, resilience and adaptability (beyond heavy reliance on prior academic attainment as the main criterion)?
• is selection for medical education fair? How do we address issues regarding widening participation and diversity in practice?
• to what extent do political, cultural and social factors influence selection philosophy and policies internationally?
• what are the risks to effective selection (e.g. access to coaching, legal challenge of poor practices) and,
• a new Ottawa consensus on selection and recruitment has been published - to what extent does this statement reflect your experiences of designing and implementing selection systems in your locality?

In contributing to the debate, this special issue provides a platform for authors to present the latest research, empirical studies, systematic reviews, reflections, case studies and practical tips on current/future issues in selection and recruitment in medical education.

## Introduction

Internationally, selection into medicine continues to be highly competitive and is considered ‘high stakes’ by a multitude of different stakeholders. As the discipline is changing rapidly, to what extent are current selection and recruitment practices aligned with educating those people most likely to be competent clinicians with the right values in the 21 ^st^ century? Practically, selection can be highly resource intensive, so what are the most effective and efficient approaches to designing robust selection methods and systems for today and tomorrow?

This special issue provides a platform to present the latest research, reflections, case studies and practical tips on current and future issues in selection and recruitment in medical education.

There is over a century of research on selection and recruitment and the field has both matured and expanded significantly over this time. Many of the same challenges remain however, and there is growing recognition that there is no ‘silver bullet’ solution, reflecting that the topic is both multi-layered and multi-faceted, where ‘one size does not fit all’. Such is the complexity, some authors argue selection to medical education is a ‘wicked problem’ ( Cleland, Patterson et al, 2018).

Previous research has tended to focus on reviewing the utility of different selection methods but now the discourse has turned to more complex issues including: selection policies; selection systems design, social accountability, diversity and fairness, and workforce shortages in some specialities (e.g. community medicine) and in certain contexts (e.g. remote and rural working, and in developing countries). In many areas there remains a significant gap between the research evidence and policy enactment across the globe.

We encourage authors to consider the following key questions and themes (amongst others):

## How may current and/or advancing technologies (e.g. social media, big data, virtual reality, artificial intelligence) influence selection methods for the future?

The increasing use of technology to manage the complexity of 21st century medicine will require fundamental changes in the way clinicians ‘think about thinking’. Obermeyer and Lee (2018) argue that today’s medical education systems are ill prepared to meet these challenges, where there is very little training in the data science, statistics, or behavioural science required for effective clinical practice in the future. Similarly, to what extent are these skills sets reflected in the selection criteria for medical school admissions?

## What are the important selection criteria for the future (e.g. creativity and innovation, resilience, adaptability, compassion) and to what extent should we/can we assess these at point of selection?

Given the speed with which patient needs and disease patterns are changing, now more than ever healthcare systems internationally require students, trainees and employees who can innovate ( Patterson & Zibarras, 2017). Economically, the ability to innovate is one of the few strategic ways organisations can be proactive in learning how to ‘do more with less’ which similarly requires attributes relating to resilience and adaptability, in order to cope effectively with increasing work demands. Despite this pressing need, few selection systems in medical education prioritise the potential for creative-problem solving, adaptability and innovation.

## To what extent is selection ‘fair’ – how can we address schools’ pursuit of prestige whilst broadening stakeholder participation, and addressing issues regarding differential attainment, diversity and improving widening access?

Fairness and diversity continue to be critically important issues in selection and recruitment practices internationally. Yet it remains unclear as to which approaches are most effective in terms of attracting, recruiting and supporting under-represented groups into medical school, through to specialty training and into practice.

Regarding socioeconomic status (SES), diversity in medical education has been hindered by the predominant and heavy reliance on indicators of prior academic attainment and/or cognitively-oriented tests as formal medical school admission tools (see Cleland, Alexander et al, 2018 for a review). Since those from lower socio-economic backgrounds tend to have less access to good quality education they are at an immediate disadvantage. Regarding ethnicity, there is similar differential attainment across sub-groups evidenced wherever one sits in the globe. Regarding gender, historically medicine was male dominated but this picture is changing rapidly where now there are at least equal numbers of women (if not more) entering medical school in many parts of the globe. However, there remain issues regarding fairness issues in selection, for example, the recent media storm surrounding claims of discrimination against women students over many years by a Japanese medical school. The Tokyo Medical University were reported to be docking the entrance scores of female applicants to keep the ratio of women-to-men at 30 percent. Media reports implied that the University manipulated entrance results through fears of later staff shortages when women start families ( Guardian, 2018).

## Recruitment and selection are often heavily influenced by the local political landscape, what can we learn from international research evidence to impact future policy and practice?

Whilst the field of selection has developed considerably over time, the majority of published research available continues to originate from a small number of global regions (Europe, North America and Australasia). Given that selection and recruitment practices are inextricably linked to the political, cultural and social context in which they are conducted, what can be learned from research in other countries (e.g. Asia, South America, the Middle East and Africa) where case material is severely lacking.

## Identifying and addressing the risks to effective selection

Many institutions are now using more ‘evidence-based’ approaches in their selection procedures (such as multiple mini-interviews and situational judgement tests). However, there is ongoing use of selection methods that have little or no research evidence supporting them (e.g. personal statements, references). Poor selection practices are at risk of legal challenge. Little previous research has adequately addressed how best to deal with the risk of susceptibility to coaching, where coaching is now a major industry (see Griffin, 2018).

## A new consensus statement

A number of these themes and challenges are discussed in a recently published international Ottawa consensus statement on selection and recruitment in healthcare ( Patterson et al, 2018). To what extent does this statement reflect your views and experiences in researching and implementing selection systems in your locality?

We invite your contribution to the debate in this special issue of MedEdPublish on selection and recruitment in medical education.

## Take Home Messages

• Selection for medical education continues to be highly competitive and is considered high stakes for all concerned.
• Good quality research has emerged on the relative effectiveness of different selection methods to inform those implementing selection. However, in many areas, there remains a significant gap between the research evidence and selection practices (e.g. use of personal statements, widening participation, diversity and fairness issues).
• Selection system design is influenced by many complex political, cultural and social factors and case material is currently lacking from some parts of the globe (e.g. Africa, Middle East, Asia, etc) to help inform the debate.
• Selection practices in future will be influenced by new developments in technology (e.g. social media, artificial intelligence) and new selection criteria should be considered (e.g. creativity, adaptability, resilience) beyond heavy reliance on prior academic attainment, as the discipline of medicine continues to evolve.

## Notes On Contributors

Professor Fiona Patterson is a founding Director of Work Psychology Group, a research-led consulting practice with specialist expertise in selection. She is a Visiting Researcher at the Universities of Cambridge, London, Nottingham and Aberdeen. She Co-Chairs an international research network for selection for the healthcare professions (INReSH) with contributors from around the globe. She led the 2018 Ottawa consensus statement on selection and recruitment in healthcare hosted by AMEE. ORCID https://orcid.org/0000-0002-1031-130X

Professor Barbara Griffin is a member of the Department of Psychology at Macquarie University and an endorsed organisational psychologist. She has led the development of selection processes for both undergraduate and postgraduate medical programs in Australia and consults to industry and specialist medical colleges on selection issues. ORCID https://orcid.org/0000-0002-3597-7351

Dr. Mark D. Hanson is a Psychiatrist at the Hospital for Sick Children and Professor, Department of Psychiatry, Faculty of Medicine, University of Toronto, Canada. Previous medical education positions held include Associate Dean/Director Admissions and Student Financial Aid, Undergraduate Medicine, Faculty of Medicine, University of Toronto. Admissions scholarship focuses upon issues of social accountability, admissions interviewing and file review methods. ORCID https://orcid.org/0000-0002-0820-4521

## Declarations

Fiona Patterson is a Director in Work Psychology Group, a research consulting practice which receives funding from Health Education England, UKCAT and other educational institutions to design and evaluate a range of selection methods and systems. Fiona is a guest Theme Editor for the theme of Selection and Recruitment in Medical Education in AMEE MedEdPublish.Mark D Hanson holds an Associated Medical Services (AMS) Phoenix Fellowship Award to examine a New Health Professions Admission Model Grounded in Patient Defined Application Attributes, Processes and Policies. Mark is a guest Theme Editor for the theme of Selection and Recruitment in Medical Education in AMEE MedEdPublish.Barbara Griffin is a guest Theme Editor for the theme of Selection and Recruitment in Medical Education in AMEE MedEdPublish.
