# Supplementary material for: Opening Editorial: Selection and Recruitment in Medical Education
Source: MedEdPublish (2016). 2018 Oct 1;7:222. [Version 1] doi: 10.15694/mep.2018.0000222.1 (PMC10704444; doi:10.15694/mep.2018.0000222.1)
Supplement: Peer review history

# Reviewer report: Reviewer response for version 1

**DOI:** 10.21956/mep.19688.r29420

**Published:** 2018-10-02

**Authors:** Trevor Gibbs

**Affiliations:** 1 AMEE

This review has been migrated. The reviewer awarded 5 stars out of 5 As the authors quite rightly say: Many challenges remain however, reflecting that selection and recruitment into medical education (both undergraduate and postgraduate) is a complex, multi-dimensional, dynamic phenomenon."Although so many challenges exist in this ever-changing world of ours, I am looking forward to this theme in the hope that it provides some answers to such a difficult and taxing situation. Maybe we will never get it completely right, simply because we are constantly running to catch up with the expectations of our future healthcare practitioners.The authors have created an excellent list of key questions and sub-themes and already provided a very helpful list of essential papers related to the topic.

Reviewer Expertise:

NA

No decision status is available

# Reviewer report: Reviewer response for version 1

**DOI:** 10.21956/mep.19688.r29422

**Published:** 2018-10-01

**Authors:** Sateesh Babu Arja

**Affiliations:** 1 Avalon University School of Medicine

This review has been migrated. The reviewer awarded 5 stars out of 5 I would like to thank all authors for the opening editorial on selection and recruitment in medical education. It is one of the challenging issues in medical education. As the authors quoted selection in medical education is a wicked problem. We always wonder how one number or GPA or performance on a particular test decides the entry of one student into medical education or post graduation training. Then we are practicing the multiple-mini interviews (MMI) and situational judgment tests (SJT). Do we measure the right traits? Are these traits stable? Do they change? Is it not the responsibility of medical education to teach these students on how to be more of a team palyer or Medical Ethics? But we assess these attributes in the selection process. Many papers are supporting MMIs and SJT. But we have an equal number of papers shows no significant difference between traditional interviews and MMI. One such paper which got my attention is Jerant A, Henderson MC, Griffin E, Hall TR, Kelly CJ, Peterson EM, Wofsy D, Franks P (2018). Do Multiple Mini-Interview and Traditional Interview Scores Differ in Their Associations With Acceptance Offers Within and Across Five California Medical Schools? Acad Med. 2018 Mar 20;. Epub 2018 Mar 20.The other issue is nowadays the private medical schools are growing day by day. Are these private medical schools are committed to diversity and serving disadvantaged socioeconomic groups or communities? The other issue that I would like to add is in a country like India, the reservations in medical education and postgraduate training programs are there based o caste system. It is a debatable topic, and we have an equal number of supporters and people who are against the reservations system. The real question is this reservation system really helps in lifting the low socioeconomic groups?. The reservation system in India has cultural and political influence. Thank you

Reviewer Expertise:

NA

No decision status is available

# Reviewer report: Reviewer response for version 1

**DOI:** 10.21956/mep.19688.r29421

**Published:** 2018-10-01

**Authors:** Leila Niemi-Murola

**Affiliations:** 1 University of Helsinki

This review has been migrated. The reviewer awarded 5 stars out of 5 Thank you for the invitation to review this interesting opening editorial. The selection criteria are a hot topic in my country, too. There is evidence indicating that the student selection favors students coming from families having academic background and high income. However, we need physicians having different backgrounds and interested in various fields of medicine. Some students mature later than others and the grades given in the high school or college might not inform us about the student's full capacity. Thus, new selection methods are more than welcome. This editorial gives us valuable new insights to student selection in the near future.

Reviewer Expertise:

NA

No decision status is available
